# Validation of EuroSCORE II in atrial fibrillation heart surgery patients from the KROK Registry

**DOI:** 10.1038/s41598-023-39983-w

**Published:** 2023-08-10

**Authors:** Łukasz Kuźma, Mariusz Kowalewski, Wojciech Wańha, Emil Julian Dąbrowski, Marek Jasiński, Kazimierz Widenka, Marek Deja, Krzysztof Bartuś, Tomasz Hirnle, Wojciech Wojakowski, Roberto Lorusso, Zdzisław Tobota, Bohdan J. Maruszewski, Piotr Suwalski, KROK Investigators, Lech Anisimowicz, Andrzej Biederman, Dariusz Borkowski, Mirosław Brykczyński, Paweł Bugajski, Marian Burysz, Paweł Cholewiński, Romuald Cichoń, Marek Cisowski, Antoni Dziatkowiak, Tadeusz Gburek, Witold Gerber, Leszek Gryszko, Ireneusz Haponiuk, Piotr Hendzel, Stanisław Jabłonka, Krzysztof Jarmoszewicz, Ryszard Jaszewski, Marek Jemielity, Ryszard Kalawski, Bogusław Kapelak, Maciej A. Karolczak, Jacek Kaperczak, Piotr Knapik, Michał Krejca, Wojciech Kustrzycki, Mariusz Kuśmierczyk, Paweł Kwinecki, Leszek Markuszewski, Maurycy Missima, Jacek J. Moll, Wojciech Ogorzeja, Jacek Pająk, Michał Pasierski, Wojciech Pawliszak, Edward Pietrzyk, Grzegorz Religa, Jan Rogowski, Jacek Różański, Jerzy Sadowski, Girish Sharma, Janusz Skalski, Jacek Skiba MD, Ryszard Stanisławski, Janusz Stążka, Sebastian Stec, Piotr Stępiński, Grzegorz Suwalski, Kazimierz Suwalski, Łukasz Tułecki, Waldemar Wierzba, Michał Wojtalik, Stanisław Woś, Michał Oskar Zembala, Piotr Żelazny

**Affiliations:** 1 Department of Invasive Cardiology, Medical University of Bialystok, Bialystok, Poland; 2 Clinical Department of Cardiac Surgery and Transplantology, National Medical Institute of the Ministry of Interior and Administration, Warsaw, Poland; 3 Thoracic Research Centre, Innovative Medical Forum, Collegium Medicum Nicolaus Copernicus University, Bydgoszcz, Poland; 4 Cardio-Thoracic Surgery Department, Heart and Vascular Centre, Maastricht University Medical Centre (MUMC), Cardiovascular Research Centre Maastricht (CARIM), Maastricht, The Netherlands; 5 Department of Cardiology and Structural Heart Diseases, Medical University of Silesia, Katowice, Poland; 6 Department and Clinic of Cardiac Surgery, Wroclaw Medical University, Wroclaw, Poland; 7 Clinical Department of Cardiac Surgery, District Hospital No. 2, University of Rzeszów, Rzeszów, Poland; 8 Department of Cardiac Surgery, Upper-Silesian Heart Center, Katowice, Poland; 9 Department of Cardiovascular Surgery and Transplantology, Jagiellonian University Medical College, John Paul II Hospital, Krakow, Poland; 10 Department of Cardiosurgery, Medical University of Bialystok, Bialystok, Poland; 11 Department of Pediatric Cardiothoracic Surgery, The Children’s Memorial Health Institute, Warsaw, Poland; 12 Department of Cardiac Surgery, University Hospital, Bydgoszcz, Poland; 13 Cardiac Surgery Department, Medicover Hospital, Warsaw, Poland; 14 Department of Cardiac Surgery, Masovian Specialistic Hospital of Radom, Radom, Poland; 15 Department of Cardiac Surgery, Pomeranian Medical University, Szczecin, Poland; 16 Department of Cardiosurgery, J. Struś Hospital, Poznań, Poland; 17 Department of Cardiac Surgery, Regional Specialist Hospital, Grudziadz, Poland; 18 Department of Cardiac Surgery, Medinet Heart Center Ltd, Wroclaw, Poland; 19 Department of Cardiovascular Surgery, University Clinical Center of the Medical University of Warsaw, Warsaw, Poland; 20 Department of Cardiac Surgery, American Heart of Poland, Bielsko-Biała, Poland; 21 Department of Cardiovascular Surgery and Transplantology, Jagiellonian University Medical College, John Paul II Hospital, Krakow, Poland; 22 Department of Cardiac Surgery, The Pope John Paul II Province Hospital, Zamość, Poland; 23 Department of Cardiac Surgery, Military Institute of Medicine, Warsaw, Poland; 24 Department of Pediatric Cardiac Surgery, Pomeranian Traumatology Center, Gdańsk, Poland; 25 Medical University of Warsaw, Warsaw, Poland; 26 Cardiac Surgery Department, Medical University of Lublin, Lublin, Poland; 27 Department of Cardiac Surgery, Ceynowa Specialist Hospital, Wejherowo, Poland; 28 Lower Silesian Center for Heart Diseases, Nowa Sól, Poland; 29 Department of Cardiac Surgery, Medical University of Lodz, Lodz, Poland; 30 Department of Cardiac Surgery and Transplantology, Poznan University of Medical Sciences, Poznań, Poland; 31 Department of Cardiac and General Pediatric Surgery, Medical University of Warsaw, Warszawa, Poland; 32 Department of Cardiac Surgery, University Hospital, Institute of Medical Sciences, University of Opole, Opole, Poland; 33 Department of Anesthesiology and Intensive Therapy, Silesian Centre for Heart Diseases, Medical University of Silesia, Zabrze, Poland; 34 Department of Cardiac Surgery, Wroclaw Medical University, Wroclaw, Poland; 35 Department of Cardiac Surgery and Transplantology, National Institute of Cardiology, Warszawa, Poland; 36 Department of Medicine, Faculty of Medical Sciences and Health Sciences, Kazimierz Pulaski University of Technology and Humanities in Radom, Radom, Poland; 37 Cardiology and Cardiac Surgery Department, 11th Military Research Hospital and Polyclinic IPHC in Bydgoszcz, Bydgoszcz, Poland; 38 Department of Cardiac Surgery, Polish Mother’s Memorial Hospital Research Institute, Lodz, Poland; 39 Clinical Department of Cardiac Surgery and Transplantology, National Medical Institute of the Ministry of Interior and Administration, Centre of Postgraduate Medical Education, Warsaw, Poland; 40 Department of Cardiac Surgery, Swietokrzyskie Cardiology Center, Kielce, Poland; 41 Department of Cardiac Surgery, Bieganski Hospital, Łódź, Poland; 42 Department of Cardiac and Vascular Surgery, Medical University of Gdansk, Gdańsk, Poland; 43 Pediatric Cardiac Surgery, Jagiellonian University, Krakow, Poland; 44 Department of Cardiac Surgery, 4th Military Hospital, Wrocław, Poland; 45 Subcarpathian Center for Cardiovascular Intervention, Sanok, Poland; 46 Department of Paediatric Cardiac Surgery, Poznan University of Medical Sciences, Poznan, Poland; 47 2nd Department of Cardiac Surgery, Medical University of Silesia, Katowice, Poland; 48 Division of Cardiac Surgery, Heart and Lung Transplantation and Mechanical Circulatory Support, Silesian Center for Heart Disease, Zabrze, Poland; 49 Department of Cardiac Surgery, Voivodeship Specialist Hospital of Olsztyn, Olsztyn, Poland

**Keywords:** Interventional cardiology, Myocardial infarction, Cardiovascular diseases, Prognosis

## Abstract

The study aimed to validate the European System for Cardiac Operative Risk Evaluation score (EuroSCORE II) in patients with atrial fibrillation (AF). All data were retrieved from the National Registry of Cardiac Surgery Procedures (KROK). EuroSCORE II calibration and discrimination performance was evaluated. The final cohort consisted of 44,172 patients (median age 67, 30.8% female, 13.4% with AF). The in-hospital mortality rate was 4.14% (N = 1830), and 5.21% (N = 2303) for 30-day mortality. EuroSCORE II significantly underestimated mortality in mild- and moderate-risk populations [Observed (O):Expected (E)—1.1, 1.16). In the AF subgroup, it performed well [O:E—0.99), whereas in the very high-risk population overestimated mortality (O:E—0.9). EuroSCORE II showed better discrimination in AF (−) [area under curve (AUC) 0.805, 95% CI 0.793–0.817)] than in AF (+) population (AUC 0.791, 95%CI 0.767–0.816), P < 0.001. The worst discriminative performance for the AF (+) group was for coronary artery bypass grafting (CABG) (AUC 0.746, 95% CI 0.676–0.817) as compared with AF (−) population (AUC 0.798, 95% CI 0.774–0.822), P < 0.001. EuroSCORE II is more accurate for patients with AF. However, it underestimated mortality rates for low-to-moderate-risk patients and had a lower ability to distinguish between high- and low-risk patients with AF, particularly in those undergoing coronary artery bypass grafting.

## Introduction

Despite the steady decline in the number of performed cardiac surgeries, every year more than 300,000 coronary artery bypass graft (CABG) and valve operations are performed in the United States^1^. Among patients undergoing cardiac surgery, atrial fibrillation (AF) prevalence is estimated for 11.5%, which makes it one of the most common co-morbidities in this group^2^. AF is often considered as an indicator of high-risk patients and a predictor of higher mortality rates and potentially fatal postoperative complications. As a consequence of loss of atrial systole contribution, greater morbidity rates for stroke and renal failure, prolonged ventilation time, higher reoperation rates, and deep sternal wound complications have been reported. Moreover, patients with pre-operative AF experience a higher adjusted long-term risk of all-cause death and cumulative risk of stroke and systemic embolism^3,4^.

The European System for Cardiac Operative Risk Evaluation (EuroSCORE) II was developed to reflect a more current dataset and evidence-based improvements in cardiac surgery. In the United States, The Society of Thoracic Surgeons (STS) risk score is more accepted owing to the relatively high predictive value despite less user friendliness and inapplicability to some cardiac surgeries. The inclusive nature of EuroSCORE II for numerous procedures provides more flexibility than the STS score for complex procedures. Unlike the STS risk score, EuroSCORE II does not include AF as a risk factor, often leading to the underestimation of risk in higher-risk-profile patients.

The current analysis aimed to validate EuroSCORE II on the robust cohort of heart surgery patients with underlying AF from a contemporary nationwide registry.

## Methods

### Registry design

All data were retrieved from the Polish National Registry of Cardiac Surgery Procedures (KROK). Data collection methods and definitions are available at https://krok.csioz.gov.pl. Initially, our data set consisted of 45,050 adult cardiac surgical patients from January, 1st 2017 to January, 1st 2020 from 37 enrolling centres. However, patients were excluded from the study if any of the following exclusion criteria were met: patients with missing > 1 EuroSCORE II predictors or with missing in-hospital mortality data (N = 512), patients aged under 18 years (N = 226) or over 90 years due to not enough data for nonagenarians in the time of creating EuroSCORE II risk model (N = 140). EuroSCORE II was recalculated for every single patient enrolled in the study with the interactive calculator (available at https://www.euroscore.org). Depending on the calculated score, EuroSCORE II situates patients at ≤ 2%—low, 2% to ≤ 5%—mild, > 5% to ≤ 10%—moderate, 10% to ≤ 20%—high, and > 20%—very high risk of perioperative in-hospital mortality. The diagnosis of any type of pre-operative AF was based on the anamnesis interview.

### Data collection

A detailed questionnaire, defined according to standard definitions, including demographic data, history, physical findings, management, imaging studies, and outcomes, was developed. Data were collected either at presentation or by physician review of the hospital records and were forwarded to the KROK registry. For patients undergoing heart surgery, we considered and reported the variables according to EuroSCORE II definitions. Additionally, exact types of surgeries were reported alongside. The study was approved by the Institutional Board of Central Clinical Hospital of the Ministry of Interior, Centre of Postgraduate Medical Education, Warsaw, Poland and adheres to Helsinki Declaration as revised in 2013. Due to the anonymization of registry data, patient informed consent was waived by the Institutional Board of Central Clinical Hospital of the Ministry of Interior, Centre of Postgraduate Medical Education, Warsaw, Poland.

The primary endpoint was in-hospital mortality as per EuroSCORE II definition, together with 30- and 90-day mortality.

### Statistical analysis

Normal distribution was assessed using a Shapiro–Wilk test. Descriptive analyses were represented as a median (Me) with interquartile range (IQR) for continuous variables, and for categorical variables as a number (N) of occurrences (%). The statistical significance of differences between the two groups was determined using the χ^2^, Mann–Whitney U and Dunn's multiple comparisons tests when appropriate. The association between mortality, EuroSCORE II and atrial fibrillation (AF) was assessed using univariable and multivariable logistic regression.

Model calibration was evaluated using the Hosmer–Lemeshow test (data collapsed into 10 quartiles of estimated probability), calibration plot methodology (predicted probability of expected (E) vs. the observed (O) proportion) of outcomes for 50 equally sized groups in two cohorts according to the presence or lack of AF. The expected mortality rate was compared with the observed mortality rate in the overall cohort and clinically defined sub-groups according to mortality risk and AF. The estimated survival probability was presented graphically by Kaplan–Meier curves^5^.

Discriminative performance was assessed by receiver operating characteristic (ROC) curves and by computing the area under the curve (AUC) with a 95% confidence interval (95% CI). The AUCs were compared using the Delong test. The tests were assessed in the overall study group and the subgroups of patients with AF regardless of the type of surgery. For all analyses, we set the level of statistical significance at P < 0.05. All statistical analysis was performed using XL Stat (Addinsoft, 2022, version 2022.1.02.1251, New York, NY, USA), and Stata Statistical Software, (StataCorp, 2022, version 17, TX, USA).

## Results

**Figure 1:**
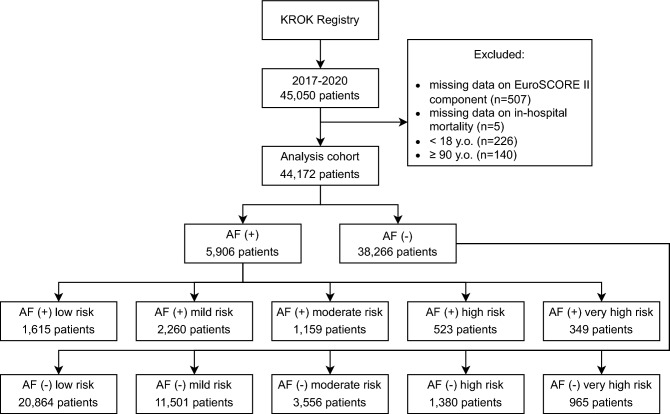
Flow chart of study design. *AF* atrial fibrillation.

The final cohort for analysis consisted of 44,172 patients (Fig. 1). The median age of the population was 67 years (IQR 60–72), 30.8% (N = 13,604) of patients were female, and 13.4% (N = 5906) had AF. Detailed characteristics with a comparison between AF (+) and AF (−) groups are shown in Table 1. Patients with AF had a higher percentage of all major complications, i.e., prolonged mechanical ventilation, surgical-site infection, bleeding, reoperation, stroke, and acute kidney injury excluding acute coronary syndromes. Overall, the in-hospital mortality rate was 4.14% (N = 1830), and 5.21% (N = 2303) for 30-day mortality (Table S1—supplementary materials).

**Table 1:** Characteristics of the screened population.

Variable	All patients (N = 44,172)	AF (+) group (N = 5906)	AF (−) group (N = 38,266)	P
Age (years); Me [IQR]	67 [60–72]	70 [64–75]	66 [60–72]	< 0.001
Female gender; % (N)	30.8 (13,604)	37.1 (2,192)	29.8 (11,412)	< 0.001
BMI; Me [IQR]	27.7 [25–31]	27.8 [24–30]	27.7 [25–31]	< 0.001
CCS class IV; % (N)	3.6 (1592)	2.8 (164)	3.7 (1428)	< 0.001
NYHA class IV; % (N)	4.8 (2106)	8.2 (487)	4.2 (1619)	< 0.001
MI within previous 90 days	26.6 (11,753)	20.2 (1195)	27.6 (10,558)	< 0.001
IDDM; % (N)	11.7 (5147)	12.2 (713)	11.6 (4434)	0.3
Extracardiac arteriopathy; % (N)	21.9 (9663)	24.9 (1469)	21.4 (8194)	< 0.001
CPD; % (N)	12.9 (5712)	10.9 (643)	13.2 (5069)	< 0.001
Dialysis; % (N)	0.9 (419)	1.5 (87)	0.9 (332)	< 0.001
eGFR < 50 ml/min/1.73 m^2^; % (N)	11.2 (4951)	18.2 (1072)	10.1 (3879)	< 0.001
Poor mobility; % (N)	8.6 (3790)	13 (768)	7.9 (3022)	< 0.001
Ejection fraction				
LVEF 31–50%; % (N)	43.5 (19,229)	49.1 (2,901)	42.7 (16,328)	< 0.001
LVEF 20–30%; % (N)	5.1 (2263)	7.9 (464)	4.7 (1799)	
LVEF ≤ 20%; % (N)	1.8 (809)	2.8 (164)	1.7 (645)	
Pulmonary hypertension				
Moderate (31–55 mmHg); % (N)	15.2 (6731)	20.5 (1209)	14.4 (5522)	< 0.001
Severe (> 55 mmHg); % (N)	1.6 (724)	4.7 (275)	1.2 (449)	
Previous cardiac surgery; % (N)	7.2 (3176)	12.4 (731)	6.4 (2445)	< 0.001
Active endocarditis; % (N)	2.7 (1182)	3.1 (186)	2.6 (996)	0.02
Critical preoperative state; % (N)	3.7 (1617)	4.4 (262)	3.5 (1335)	< 0.001
Type of surgery				
Single non-CABG; % (N)	35.9 (15,857)	40.9 (2417)	35.1 (13,440)	< 0.001
2 procedures; % (N)	13.5 (5984)	22.6 (1334)	12.2 (4650)	
3 procedures; % (N)	3.8 (1657)	14 (824)	2.23 (833)	
Surgery on thoracic aorta; % (N)	8.5 (3749)	7 (415)	8.7 (3334)	< 0.001
Urgency of operation				
Urgent; % (N)	27.9 (12,340)	25.3 (11,496)	28.3 (10,844)	< 0.001
Emergency; % (N)	3.7 (1648)	3.1 (181)	3.8 (1467)	
Salvage; % (N)	1.2 (518)	1 (61)	1.2 (457)	
EuroSCORE II; Me [IQR]	2 [1.2–3.8]	3.4 [1.9–6.6]	1.8 [1.1–3.4]	< 0.001

*AF* atrial fibrillation, *BMI* body mass index, *CABG* coronary artery bypass surgery, *CCS* Canadian Cardiovascular Society grading of angina pectoris, *CPD* chronic pulmonary disease, *eGFR* estimated glomerular filtration rate, *IDDM* insulin-dependent diabetes mellitus, *IQR* interquartile range, *LVEF* left ventricular ejection fraction, *Me* median, *MI* myocardial infarction, *N* number, *NYHA* New York Heart Association Functional Classification.

The median hospital length of stay in the overall population was 3.7 days, (IQR 0.6–13.7 days). Comparing alive and in hospital died patients, those who died were older [69 (62–75) vs. 66 (60–75) years, P < 0.001], more often women [37.9% (N = 694) vs. 30.5% (N = 12,910), P < 0.001], and more often had atrial fibrillation [20.4 (N = 374) vs. 13.1% (N = 5532), P < 0.001]. Their EuroSCORE II was higher [7.0 (3.1–26) vs. 1.9 (1.2–3.5), P < 0.001] with significant differences in all parameters included in the EuroSCORE II model. (Table S2—supplementary materials). The detailed list of performed procedures is presented in Table S3.

### Association with in-hospital mortality

In univariable analysis, EuroSCORE II was associated with in-hospital mortality with an unadjusted odds ratio (OR) of 1.38 (95% CI 1.36–1.4), P < 0.001. Similarly, AF was associated with mortality with an unadjusted OR of 1.71 (1.52–1.92), P < 0.001. In a multivariable analysis, with all the variables included in EuroSCORE II scale, AF was not associated with in-hospital mortality anymore (OR 0.99, 95% CI 0.86–1.14, P = 0.87) (Table S4—supplementary materials). The multivariable analysis of EuroSCORE II thresholds showed no statistically significant impact of AF on the frequency of hospital mortality, although the impact of AF was numerically most pronounced in the highest risk groups (≥ 10%, Table S5—supplementary materials).

**Figure 2:**
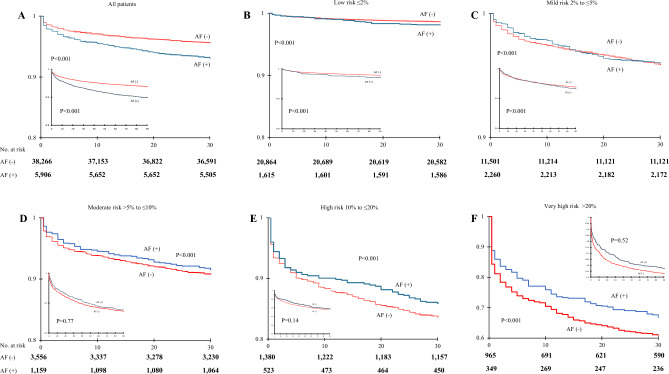
Kaplan–Meier 30-days (inner graphs—90-days) survival analysis in relation to perioperative risk and atrial fibrillation (AF). Patients are stratified to: (**A**) total population; (**B**) low-risk patients; (**C**) mild risk patients; (**D**) moderate risk patients; (**E**) high risk patients. Significant differences between occurrence of AF and survival are evident in all groups in 30-days follow-up (p < 0.001) and in patients with risk ≤ 5% in 90-days follow-up.

The Kaplan–Meier survival analysis showed a significantly higher 30 days mortality rate in the AF (+) group compared to the patients without AF (P < 0.001) but the effect is only expressed in patients with EuroSCORE II less than 5% (Fig. 2 and Table S6).

**Figure 3:**
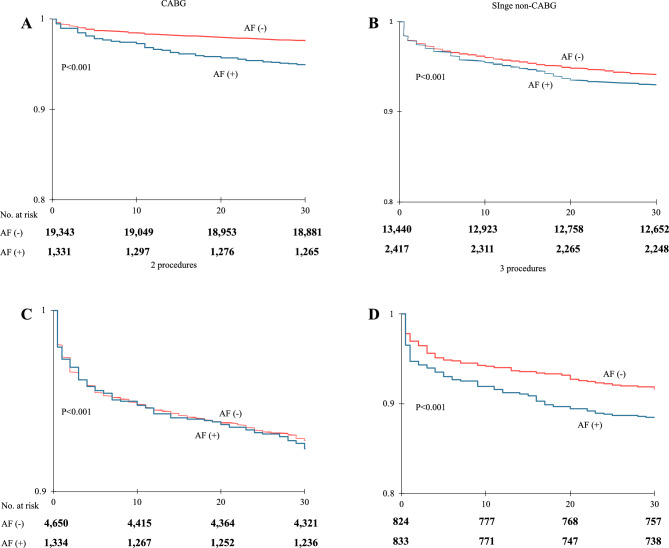
Kaplan–Meier 30-days survival analysis in relation to type of surgery and atrial fibrillation. (**A**) Coronary artery bypass grafting (CABG); (**B**) single non-CABG, (**C**) 2-procedures; (**D**) 3-procedures. Significant differences between occurrence of AF and survival are evident in all groups (p < 0.001).

In survival analysis in relation to the type of surgery and AF, the highest differences in mortality were observed in CABG and three surgery procedures as opposed to single non-CABG and two surgery procedures (Fig. 3).

### Model calibration

**Figure 4:**
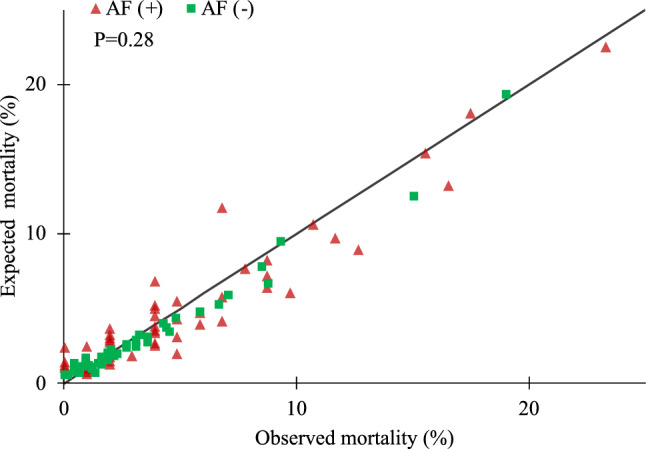
Calibration plot, comparison between observed mortality and mortality predicted by EuroSCORE II. *AF* atrial fibrillation.

The clinical performance of EuroSCORE II was tested in different populations of predicted mortality risk patients. The overall population was divided into quintiles according to EuroSCORE II and a comparison between observed and predicted in-hospital mortality according to the five models considered was made. In the total cohort, EuroSCORE II expected mortality rate was 4.01%, giving an observed to expected (O:E) ratio of 1.03. We observed under prediction of mortality for mild, moderate, and high-risk patients (O:E—1.1, 1.16, and 1.04 respectively) in opposite to low and very high-risk patients (O:E—0.91 and 0.96 respectively). In the AF (+) subgroup EuroSCORE II score performed well (O:E—0.99), whereas in the low and very high-risk populations we observed the greatest overestimation of mortality (O:E—0.89 and 0.9). On the other hand, the biggest underprediction was observed in mild and moderate-risk patients in AF (−) subgroup (O:E 1.11 and 1.18, respectively). Detailed characteristics are shown in Table S7. Visual representation of the calibration plot for AF (+) and AF (−) patients demonstrates overprediction of mortality of the EuroSCORE II model for the low and very high-risk patients as shown in Fig. 4 and Table 2.

**Table 2:** Calibration parameters in subgroups according to EuroSCORE risk and absence of atrial fibrillation.

EuroSCORE II risk		Observed mortality rate (% and 95% CI)	Expected mortality rate (% and 95% CI)	O:E ratio	O:E dif	P
All patients	Low (N = 22,479)	1.09 (0.96–1.24)	1.21 (1.2–1.21)	0.91	−0.11	0.12
	Mild (N = 13,761)	3.39 (3.08–3.68)	3.09 (3.07–3.1)	1.1	0.29	0.053
	Moderate (N = 4715)	8.02 (7.24–8.79)	6.8 (6.84–6.92)	1.16	1.14	0.004
	High (N = 1903)	14.19 (12.62–14.76)	13.69 (13.57–13.8)	1.04	0.5	0.54
	Very high (N = 1314)	35.69 (33.09–38.29)	37.27 (36.39–38.15)	0.96	−1.6	0.26
	Total (44,172)	4.14 (3.96–4.34)	4.01 (3.94–4.08)	1.03	0.14	0.2
AF (+) group	Low (N = 1615)	1.17 (0.65–1.7)	1.31 (1.29–1.33)	0.89	−0.14	0.6
	Mild (N = 2260)	3.32 (2.58–4.06)	3.24 (3.2–3.27)	1.03	0.08	0.8
	Moderate (N = 1159)	7.85 (6.3–9.4)	6.92 (6.84–7)	1.13	0.93	0.2
	High (N = 523)	13.58 (10.63–16.51)	13.65 (13.41–13.88)	0.99	−0.07	0.9
	Very high (N = 349)	33.81 (28.82–38.8)	37.47 (35.63–39.3)	0.9	−3.66	0.2
	Total (N = 5906)	6.33 (5.7–7.0)	6.38 (6.13–6.62)	0.99	0.07	0.9
AF (−) group	Low (N = 20,864)	1.09 (0.95–1.23)	1.2 (1.19–1.2)	0.91	−0.11	0.14
	Mild (N = 11,501)	3.4 (3.07–3.73)	3.06 (3.04–3.07)	1.11	0.34	0.04
	Moderate (N = 3556)	8.07 (7.18–8.97)	6.87 (6.82–6.91)	1.18	1.2	0.01
	High (N = 1380)	14.42 (12.57–16.27)	13.71 (13.56–13.85)	1.05	0.71	0.45
	Very high (N = 965)	36.37 (33.33–39.41)	37.2 (36.2–13.85)	0.98	−0.82	0.61
	Total (N = 38,266)	3.8 (3.61–3.99)	3.64 (3.58–3.71)	1.05	0.17	0.1

Patients of ≤ 2%, 2% to ≤ 5%, > 5% to ≤ 10%, 10% to ≤ 20%, and > 20% were defined to be at low, mild, moderate, high, and very high perioperative risk, respectively.

*AF* atrial fibrillation, *CI* confidence interval, *dif* difference in percentage points, *E* expected, *O* observed.

All models failed the Hosmer–Lemeshow tests (χ^2^ = 444.6, 62.7, and 363.2 for the total cohort, AF (+), and AF (−) groups, respectively. P-values were < 0.001 for all groups (Table S6).

### ROC analysis

**Figure 5:**
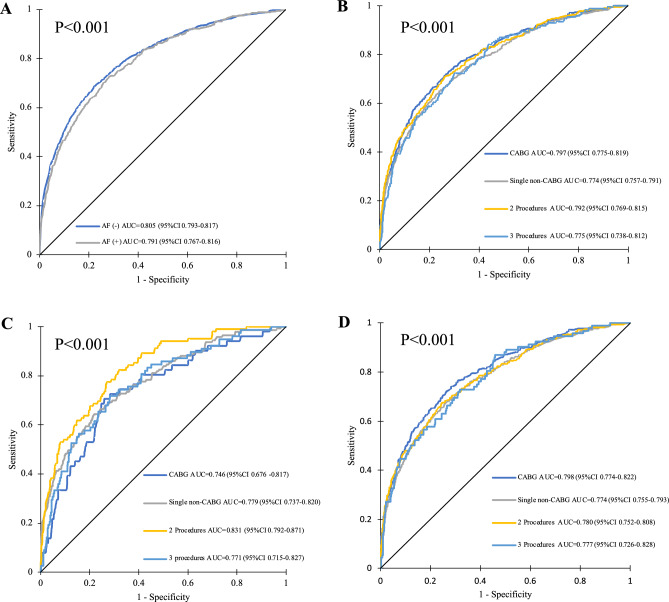
Receiver operating characteristic curves: (**A**) AF (+) vs. AF (−); (**B**) studied population for different in-hospital mortality risks; (**C**) AF (+) for different in-hospital mortality risks. (**D**) AF (−) for different in-hospital mortality risks. P for comparison between AF (−) vs. AF (+) in —CABG group (< 0.001), —singe non-CABG (0.16), —2 procedures (0.01), —3 procedures (0.44). *AUC* area under curve, *CI* confidence interval.

EuroSCORE II showed good discrimination in the overall population with the AUC of 0.807 (95% CI 0.796–0.817). The ROC curves for AF (+) group presented a lower AUC (0.791, 95%CI 0.767–0.816) than in AF (−) (AUC 0.805, 95% CI 0.793–0.817), P < 0.001 (Fig. 5).

**Figure 6:**
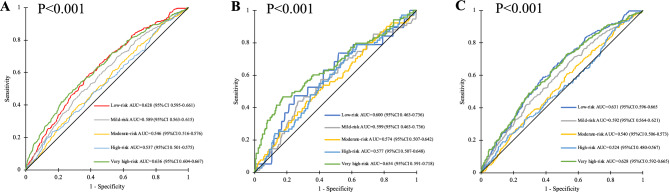
Receiver operating characteristic curves: (**A**) studied population for different in-hospital mortality risks; (**B**) AF (+) population for different in-hospital mortality risks; (**C**) AF (−) population for different in-hospital mortality risks. P for comparison between AF (−) vs. AF (+) in —low group (< 0.001), —mild (< 0.001), —moderate (< 0.001), —high (< 0.001), —very high (0.01). *AUC* area under curve, *CI* confidence interval.

The best discriminative performance of EuroSCORE II models for the AF (+) cohort was 2 procedures surgery (AUC = 0.831, 95%CI 0.792–0.871). On the other hand, the worst discriminative power of EuroSCORE II for the AF (+) was for CABG (AUC 0.746, 95%CI 0.676–0.817) as compared with AF (−) population (AUC 0.798, 95% CI 0.774–0.822), P < 0.001. Discrimination for different in-hospital mortality risk groups for the overall cohort and AF subgroup are presented in Fig. 6.

## Discussion

The current analysis is the first to perform a validation of EuroSCORE II for patients with underlying AF and undergoing heart surgery on such a scale. The main finding of the current work is that in the overall population, EuroSCORE II has good predictive value, however, its calibration was better in patients with concomitant AF. Moreover, EuroSCORE II significantly underestimated mortality in mild and moderate groups of patients. On the other hand, mortality was numerically overestimated in low and very high-risk groups, particularly in patients with AF. In this group, the model had poor performance for isolated coronary artery bypass graft surgery risk stratification and in both the highest and lowest risk patients. Therefore, EuroSCORE should be used with caution in these groups of patients.

A number of risk calculators are available to estimate the risk of mortality and complications following heart surgery both in-hospital and long-term^6^. EuroSCORE II has been accepted and widely adopted for numerous reasons—ease, readiness, and bedside use being the most important. A recent review of external validations of cardiovascular clinical prediction models (CPM) reported that EuroSCORE II is the third most validated CPM. The median AUC of 65 validations was 0.76 (IQR 0.68–0.81), proving its good discriminative performance^7^. When compared to other risk calculators, in many analyses that investigated reliability in predicting perioperative mortality in cardiac surgery patients, EuroSCORE II has provided similar results to STS score, both outperforming EuroSCORE I and ACEF (age, creatinine, ejection fraction)^6,8^. Interestingly, in the aforementioned CPMs review by Wessler et al., EuroSCORE II was found to have better discriminative power than STS score and ACEF but not EuroSCORE I^7^.

However, EuroSCORE II has its shortcomings. It was reported that the model may not be reliable in non-elective surgeries and patients undergoing valvular interventions^9–13^. Grant et al. in their analysis of 3,343 emergency procedures found that the risk tended to be underpredicted in lower-risk patients and over-predicted in the higher-risk^10^. It raised a concern that a situation in which a patient is denied an emergency cardiac surgery due to an inappropriately high-risk score can occur. On the other hand, Paparella et al., in their external validation in a prospective registry, reported that in urgent and emergent surgery observed-to-expected mortality rates were 1.43 and 1.45, respectively, suggesting significant underestimation in such cases^11^. However, both studies were consistent when considering good overall prediction of in-hospital mortality in non-emergent cases. Our results are contrary to other studies that reported underestimation of the expected mortality rates among low- and high-risk patients^11,14,15^. Moreover, our study does not support the current data suggesting well calibration of EuroSCORE II among patients with mild or moderate risk^11,16^. There may be several sources of these discrepancies. Firstly, our procedural characteristics differ from the EuroSCORE II and validation studies reports. The main differences regard higher rates of combined surgeries and differences in rates of valvular interventions. On the other hand, being aware of the risk of multicollinearity and overfitting, it was proved in EuroSCORE I that the limitation of included variables resulted in better calibration and clinical performance^17^. Finally, EuroSCORE II was based on data from 43 countries, including 16 non-European. Knowing the differences in quality of care, comorbidities, and risk profiles between nations, its heterogeneity may have affected the accuracy of estimations and the results may not be generalizable to all populations^13^.

Pre- and post-operative AF is a well-known risk factor for adverse short- and long-term outcomes, including higher mortality, for both cardiac and non-cardiac surgery^4,18–20^. Not only did our study prove that AF is associated with higher peri-operative mortality rates, but also that such patients were significantly more susceptible to all of the analyzed complications, except acute coronary syndrome. The lower risk of myocardial infarction among patients with pre-operative AF is consistent with the recent Prasada et al. impressive analysis of 8,635,758 individuals who underwent non-cardiac surgery^19^. Several mechanisms explaining the detrimental effect of AF in the peri-operative period are proposed, including, among others, low-cardiac-output syndrome and impaired bypass graft flow^21^. The meta-analysis of 35 studies revealed that perioperative AF was associated with an increased risk of stroke and mortality, both in the short- and long-term^3^. In addition, AF may contribute to the development of heart failure, its exacerbations, and increased bleeding events due to the necessity for chronic oral anticoagulation^22^.

One of the important novel findings of our study is that EuroSCORE II provided even better prediction in the cohort of patients suffering from AF. However, its discriminative power was lower in this group, reaching the lowest value in isolated CABG surgery. In Kaplan–Meier 30-day survival analysis in relation to perioperative risk, there was a cross-over of mortality curves at the threshold of EuroSCORE II 5%, which may reflect differences in calibration between the two groups. Potential explanation includes the fact that in the group of patients without AF there was a higher rate of urgent, emergency and salvage operations—known for the increased peri-operative risk. Moreover, EuroSCORE II was reported to underestimate operative risk in non-elective cases, which may be reflected in the underprediction in patients with EuroSCORE II ≥ 5% in our study^11^. The observed differences were no longer significant in the long-term follow-up.

When it comes to the survival analysis in relation to the type of surgery, AF significantly worsened the prognosis in all of the analyzed procedures, which is consistent with the higher prevalence of peri-operative complications. The worst outcomes were reported for CABG and curves diverged after 10 days, which may be partially explained by the previously proposed influence of AF on early graft failure. Surgical ablation should be considered in such cases, as a significant improvement in prognosis was previously reported, especially in lower-risk patients^23^.

Our study proves that in the group of patients with AF EuroSCORE II overestimated mortality in low- and very-high-risk patients. Its discriminative power is significantly lower in the group of patients with AF, particularly those undergoing CABG. Future efforts in the development of EuroSCORE III should focus on taking into consideration minimally invasive approaches in cardiac surgery, e.g., transcatheter aortic valve implantation (TAVI), off-pump coronary artery bypass (OPCAB) or minimally invasive mitral valve surgery (MIMVS). There are also a few more alarming outcomes that require further investigation. This analysis demonstrated that AF is not a benign co-morbidity, but a serious condition that deeply affects prognosis after cardiac procedures. In the future, more emphasis should be placed on research focusing on the prevention of the most common complications. With epicardial left atrial appendage closure and the Cox-maze ablation, modern surgery offers effective treatment options that may improve short- and long-term outcomes in patients with concomitant AF.

### Limitations

Limitations of EuroSCORE II scoring system are inherent and translatable to the current analysis as well. EuroSCORE II by initially not including predefined factors such as neurological condition, blood panel counts, BMI, race, level of coronary stenosis etc. makes the general accuracy of the model lower in the specific subsets of patients underrepresented in the initial EuroSCORE II study cohort. One limitation of the current analysis is the lack of detailed information on AF in the KROK registry—we could not stratify patients depending on the type of AF (paroxysmal vs permanent) nor on the AF duration and association of outcomes with the type, doses, duration and adherence to OAC. Second, differences in the protocols and patient management, particularly during intensive care unit (ICU) stay, exist across participating centres. We have made an attempt to minimize the institutional bias by placing the time-frames of the study to best represent the contemporary surgical and ICU practice, yet not to overlap with COVID—19 pandemic which has made an early diagnosis and access to heart surgery care more difficult in the recent 3 years. However, it resulted in the inclusion a of relatively small group of high and very high-risk patients. Third, the current analysis does not assess the long-term outcomes; such an analysis could shed further light on the impact of initial EuroSCORE II on out-of-hospital outcomes as well. Finally, in our analysis all models failed the Hosmer–Lemeshow tests. Knowing concerns linked with the Hosmer–Lemeshow test, we decided to use also calibration plots in our analysis as well.

## Conclusions

The main findings of this study are that while EuroSCORE II is a good predictor of outcomes for the general population, it is more accurate for patients with concomitant AF. However, EuroSCORE II underestimated mortality rates for patients with low-to-moderate risk. Additionally, its ability to distinguish between high- and low-risk patients was lower for those with AF, especially those undergoing coronary artery bypass grafting, indicating that its use should be cautiously used in these groups.

### Supplementary Information

Supplementary Tables.

## Data Availability

The datasets used and analysed during the current study are available from the corresponding author on reasonable request.

## Abbreviations

ACEF: Age, creatinine and ejection fraction
AF: Atrial fibrillation
CABG: Coronary artery bypass surgery
EuroSCORE II: European System for Cardiac Operative Risk Evaluation II
KROK: Polish National Registry of Cardiac Surgery Procedures
STS: The Society of Thoracic Surgeons
