# Supplementary material for: Validation of EuroSCORE II in atrial fibrillation heart surgery patients from the KROK Registry
Source: Sci Rep. 2023 Aug 10;13:13024. doi: 10.1038/s41598-023-39983-w (PMC10415263; doi:10.1038/s41598-023-39983-w)
Supplement: Supplementary file 1 — Supplementary Tables. [file 41598_2023_39983_MOESM1_ESM.docx]

Validation of EuroSCORE II in atrial fibrillation heart surgery patients from the KROK Registry

Supplementary material

**KROK Investigators:**

Lech Anisimowicz MD, PhD ^1^; Andrzej Biederman MD, PhD ^2^; Dariusz Borkowski MD ^3^; Mirosław Brykczyński MD, PhD ^4^; Paweł Bugajski MD, PhD ^5^; Marian Burysz ^6^, Paweł Cholewiński ^3^; Romuald Cichoń MD, PhD ^7,8^; Marek Cisowski MD, PhD ^9^; Antoni Dziatkowiak MD, PhD ^10^; Tadeusz Gburek MD ^11^; Witold Gerber MD ^9^; Leszek Gryszko MD ^12^; Ireneusz Haponiuk MD ^13^; Piotr Hendzel MD, PhD ^14^; Stanisław Jabłonka MD, PhD ^15^; Krzysztof Jarmoszewicz MD ^16^; Ryszard Jaszewski MD, PhD ^18^; Marek Jemielity MD, PhD ^19^; Ryszard Kalawski MD, PhD ^5^; Bogusław Kapelak MD, PhD ^10^; Maciej A. Karolczak MD, PhD ^20^; Jacek Kaperczak MD ^21^; Piotr Knapik MD, PhD ^22^; Michał Krejca MD, PhD ^18^; Wojciech Kustrzycki MD, PhD ^23^; Mariusz Kuśmierczyk MD, PhD ^8^; Paweł Kwinecki MD ^7^; Leszek Markuszewski MD, PhD ^25^; Maurycy Missima MD ^26^; Jacek J Moll MD, PhD ^27^; Wojciech Ogorzeja MD ^6^; Jacek Pająk MD ^20^; Michał Pasierski ^28^;Wojciech Pawliszak MD ^1^; Edward Pietrzyk MD ^29^; Grzegorz Religa MD ^30^; Jan Rogowski MD, PhD ^31^; Jacek Różański MD, PhD ^24^; Jerzy Sadowski MD, PhD ^10^; Girish Sharma MD ^7^; Janusz Skalski MD, PhD ^32^; Jacek Skiba MD ^33^; Ryszard Stanisławski MD ^17^; Janusz Stążka MD, PhD ^15^; Sebastian Stec MD, PhD ^34^; Piotr Stępiński MD ^17^; Grzegorz Suwalski MD ^12^; Kazimierz Suwalski MD, PhD ^12^; Łukasz Tułecki MD ^11^; Waldemar Wierzba MD ^12^; Michał Wojtalik MD, PhD ^35^; Stanisław Woś MD, PhD ^36^; Michał Oskar Zembala MD, PhD ^37^ and Piotr Żelazny MD ^38^.

^1^ Department of Cardiac Surgery, University Hospital, Bydgoszcz, Poland

^2^ Cardiac Surgery Department, Medicover Hospital, Warsaw, Poland

^3^ Department of Cardiac Surgery Masovian Specialistic Hospital of Radom, Radom, Poland

^4^ Department of Cardiac Surgery, Pomeranian Medical University, Szczecin, Poland

^5^ Department of Cardiosurgery, J. Struś Hospital, Poznań, Poland

^6^ Department of Cardiac Surgery, Regional Specialist Hospital, Grudziadz, Poland

^7^ Department of Cardiac Surgery, Medinet Heart Center Ltd, Wroclaw, Poland

^8^ Department of Cardiovascular Surgery, University Clinical Center of the Medical University of Warsaw, Poland

^9^ Department of Cardiac Surgery, American Heart of Poland, Bielsko-Biała, Poland

^10^ Department of Cardiovascular Surgery and Transplantology, Jagiellonian University Medical College, John Paul II Hospital, Krakow, Poland

^11^ Department of Cardiac Surgery, The Pope John Paul II Province Hospital, Zamość, Poland

^12^ Department of Cardiac Surgery, Military Institute of Medicine, Warsaw, Poland

^13^ Department of Pediatric Cardiac Surgery, Pomeranian Traumatology Center, Gdańsk, Poland

^14^ Medical University of Warsaw, Warsaw, Poland.

^15^ Cardiac Surgery Department, Medical University of Lublin, Lublin, Poland

^16^ Department of Cardiac Surgery, Ceynowa Specialist Hospital in Wejherowo, Poland

^17^ Lower Silesian Center for Heart Diseases, Nowa Sól, Poland

^18^ Department of Cardiac Surgery, Medical University of Lodz, Lodz, Poland

^19^ Department of Cardiac Surgery and Transplantology, Poznan University of Medical Sciences

^20^ Department of Cardiac and General Pediatric Surgery, Medical University of Warsaw, Warszawa, Poland

^21^ Department of Cardiac Surgery, University Hospital, Institute of Medical Sciences, University of Opole, Opole, Poland

^22^ Department of Anesthesiology and Intensive Therapy, Silesian Centre for Heart Diseases in Zabrze, Medical University of Silesia, Poland

^23^ Department of Cardiac Surgery, Wroclaw Medical University, Wroclaw, Poland

^24^ Department of Cardiac Surgery and Transplantology, National Institute of Cardiology, Warszawa, Poland

^25^ Department of Medicine, Faculty of Medical Sciences and Health Sciences, Kazimierz Pulaski University of Technology and Humanities in Radom, Radom, Poland

^26^ Cardiology and Cardiac Surgery Department, 11th Military Research Hospital and Polyclinic IPHC in Bydgoszcz, Bydgoszcz, Poland

^27^ Department of Cardiac Surgery, Polish Mother's Memorial Hospital Research Institute, Lodz, Poland

^28^ Clinical Department of Cardiac Surgery and Transplantology, National Medical Institute of the Ministry of Interior and Administration, Centre of Postgraduate Medical Education, Warsaw, Poland

^29^ Department of Cardiac Surgery, Swietokrzyskie Cardiology Center, Kielce, Poland

^30^ Department of Cardiac Surgery, Bieganski Hospital, Łódź, Poland

^31^ Department of Cardiac and Vascular Surgery, Medical University of Gdansk, Gdańsk, Poland

^32^ Pediatric Cardiac Surgery, Jagiellonian University, Krakow, Poland

^33^ Department of Cardiac Surgery, 4th Military Hospital, Wrocław, Poland

^34^ Subcarpathian Center for Cardiovascular Intervention, Sanok, Poland

^35^ Department of Paediatric Cardiac Surgery, Poznan University of Medical Sciences, Poznan, Poland

^36^ 2nd Department of Cardiac Surgery, Medical University of Silesia, Katowice, Poland

^37^ Division of Cardiac Surgery, Heart and Lung Transplantation and Mechanical Circulatory Support, Silesian Center for Heart Disease, Zabrze, Poland

^38^ Department of Cardiac Surgery, Voivodeship Specialist Hospital of Olsztyn, Olsztyn, Poland

Table S1. Comparison of perioperative complications in screened population with comparison between patients with and without atrial fibrillation.

| Variable | All patients  (N=44,172) | AF (+) group  (N=5,906) | AF (-) group  (N=38,266) | P |
| --- | --- | --- | --- | --- |
| Non-complication | 79.7 (35,185) | 73.1 (4,317) | 80.7 (30,868) | <0.001 |
| Prolonged mechanical ventilation | 4.2 (1,874) | 7.7 (455) | 3.7 (1,419) | <0.001 |
| Surgical-site infection; % (N) | 2.1 (910) | 2.5 (147) | 2 (763) | <0.001 |
| Bleeding; % (N) | 5.6 (2,495) | 8 (475) | 5.3 (2,020) | <0.001 |
| Return to the operating room; % (N) | 5.04 (2,225) | 6.9 (411) | 4.7 (1,814) | <0.001 |
| Acute kidney injury; % (N) | 3.0 (1,306) | 5.6 (329) | 2.6 (977) | <0.001 |
| Dialysis; % (N) | 0.9 (438) | 1.8 (104) | 0.9 (334) | <0.001 |
| Stroke; % (N) | 2 (890) | 3.1 (181) | 1.6 (709) | <0.001 |
| Acute coronary syndrome; % (N) | 7.9 (3,490) | 3.2 (189) | 8.6 (3,301) | <0.001 |
| In-hospital mortality, % (N) | 4.14 (1,830) | 6.3 (374) | 3.8 (1,456) | <0.001 |
| 30-day mortality, % (N) | 5.21 (2,303) | 7.9 (468) | 4.8 (1,835) | <0.001 |

AF, atrial fibrillation

Table S2. Comparison of in hospital died and alive patients

| Variable | Alive patients  (N=42,342) | Died in hospital (N=1,830) | P |
| --- | --- | --- | --- |
| Age (years); Me [IQR] | 69 [62-75] | 66 [60-75] | <0.001 |
| Female gender; % (N) | 30.5 (12,910) | 37.9 (694) | <0.001 |
| BMI; Me [IQR] | 27.7 [24.6-31] | 27 [24-31] | <0.001 |
| CCS class IV; % (N) | 3.5 (1,474) | 6.4 (118) | <0.001 |
| NYHA class IV; % (N) | 3.8 (1,618) | 26.7 (488) | <0.001 |
| MI within previous 90 days | 26.5 (11,215) | 29.4 (538) | 0.006 |
| Atrial fibrillation; % (N) | 13.1 (5,532) | 20.4 (374) | <0.001 |
| IDDM; % (N) | 11.5 (4,855) | 16 (292) | <0.001 |
| Extracardiac arteriopathy; % (N) | 21.4 (9,057) | 33.1 (606) | <0.001 |
| CPD; % (N) | 12.7 (5,375) | 18.4 (337) | <0.001 |
| Dialysis; % (N) | 0.8 (346) | 4 (73) | <0.001 |
| eGFR < 50 ml/min/1.73 m2; % (N) | 10.6 (4,481) | 25.7 (470) | <0.001 |
| Poor mobility; % (N) | 7.9 (3,336) | 24.8 (454) | <0.001 |
| LVEF <30%; % (N) | 6.4 (2,720) | 19.2 (352) | <0.001 |
| Pulmonary hypertension; % (N) | 16.3 (6,894) | 30.7 (561) | <0.001 |
| Previous cardiac surgery; % (N) | 6.9 (2,923) | 13.8 (253) | <0.001 |
| Active endocarditis; % (N) | 2.4 (1,011) | 9.3 (171) | <0.001 |
| Critical preoperative state; % (N) | 2.7 (1,151) | 25.5 (466) | <0.001 |
| Weight of procedure | | | |
| –CABG; % (N) | 47.8 (20,244) | 23.5 (430) | <0.001 |
| –single non-CABG; % (N) | 35.5 (15,046) | 44.3 (811) |  |
| –2 procedures; % (N) | 13.1 (5,565) | 22.9 (419) |  |
| –3 procedures; % (N) | 3.5 (1,487) | 9.3 (170) |  |
| Surgery on thoracic aorta; % (N) | 8 (3,386) | 19.8 (363) | <0.001 |
| Urgency of operation | | | |
| –elective, % (N) | 68.3 (28,948) | 39.2 (718) | <0.001 |
| –urgent; % (N) | 27.8 (11,750) | 32.2 (590) |  |
| –emergency; % (N) | 3.2 (1,335) | 17.1 (313) |  |
| –salvage; % (N) | 0.7 (309) | 11.4 (209) |  |
| EuroSCORE II; Me [IQR] | 1.9 [1.2-3.5] | 7 [3.1-26] | <0.001 |

CABG, coronary artery bypass surgery; CCS, Canadian Cardiovascular Society grading of angina pectoris; CPD, chronic pulmonary disease; eGFR, estimated glomerular filtration rate; IDDM, insulin-dependent diabetes mellitus; IQR: interquartile range; left ventricular ejection fraction; Me, median; MI, myocardial infraction; N, number; NYHA, New York Heart Association Functional Classification

Table S3. Detailed list of performed procedures.

| All patients (N=44,172) | | | |
| --- | --- | --- | --- |
| CABG; % (N)  46.8 (20,674) | | | |
| ECC | | | 52.3 (10,816) |
| OPCAB | | | 40.7 (8,413) |
| MIDCAB | | | 4.6 (956) |
| Other | | | 2.4 (489) |
|  | | TAR, % CABG (N) | 21.5 (4,340) |
| Single non-CABG; % (N)  35.9 (15,857) | | | |
| AVR, % (N) | | | 52.1 (8,265) |
| MVS, % (N) | | | 11.9 % (1,893) |
|  | MIMVS, % MVS (N) | | 28.7 (544) |
|  | MIMV Repair, % MVS (N) | | 39.5 (747) |
|  | MV Stenosis, % MVS (N) | | 9.8 (185) |
| TV Surgery, % (N) | | | 1.1% (182) |
| PV Surgery, % (N) | | | 0.1 (18) |
| AAS, % (N) | | | 9.5 (1,509) |
| Other | | | 25.2 (3,990) |
| 2 procedures; % (N)  13.5 (5,984) | | | |
| CABG+AVR, % (N) | | | 33.5 (2,007) |
| CABG+MVS, % (N) | | | 32.7 (1,955) |
|  | | TAR, % CABG+AVR/MVS (N) | 12.4 (498) |
| MVS+TVS, % (N) | | | 11.1 (663) |
| AVR+MVS, % (N) | | | 6 (357) |
| Other, % (N) | | | 16.7 (1,002) |
| 3 procedures; % (N)  3.8 (1,657) | | | |
| CABG+MVS+TVS, % (N) | | | 53.6 (888) |
| CABG+MVS+AVR, % (N) | | | 30.2 (500) |
|  | | TAR, % CABG+MVS+AVR/TVS (N) | 11 (153) |
| Other, % (N) | | | 16.2 (269) |

AAS, aortic aneurysm surgery; AVR, aortic valve replacement; CABG, coronary artery bypass surgery; ECC, extracorporeal circulation; MIDCAB, minimally invasive direct coronary artery bypass; MIMVS, minimally invasive mitral valve surgery; MV, mitral valve; MVR, mitral valve replacement; MVS, mitral valve surgery; OPCAB, off pump coronary artery bypass; PV, pulmonary valve; TAR, total arterial revascularization; TV, tricuspid valve; TVS, tricuspid valve surgery;

Table S4. Risk factors by multivariate regression model

| Risk factors | OR | Standard error | Lower 95% CI | Upper 95% CI | P |
| --- | --- | --- | --- | --- | --- |
| Atrial fibrillation | 0.989 | 0.07 | 0.86 | 1.136 | 0.87 |
| Age (years) | 1.02 | 9.003 | 1.015 | 1.026 | <0.001 |
| NYHA class II | 0.727 | 0.054 | 0.63 | 0.84 | <0.001 |
| NYHA class III | 0.943 | 0.077 | 0.803 | 1.107 | 0.47 |
| NYHA class IV | 1.396 | 0.15 | 1.131 | 1.723 | 0.002 |
| CCS class IV | 0.98 | 0.125 | 0.763 | 1.26 | 0.9 |
| IDDM | 1.302 | 0.097 | 1.125 | 1.507 | <0.001 |
| Female gender | 1.365 | 0.078 | 1.221 | 1.527 | <0.001 |
| Extracardiac arteriopathy | 1.417 | 0.086 | 1.258 | 1.597 | <0.001 |
| CPD | 1.554 | 0.114 | 1.345 | 1.795 | <0.001 |
| Poor mobility | 1.251 | 0.099 | 1.071 | 1.469 | 0.01 |
| Previous cardiac surgery | 1.424 | 0.118 | 1.211 | 1.676 | <0.001 |
| Dialysis | 3.411 | 0.603 | 2.413 | 4.823 | <0.001 |
| eGFR < 50 ml/min/1.73 m2 | 1.788 | 0.138 | 1.536 | 2.079 | <0.001 |
| eGFR 50-85 ml/min/1.73 m2 | 1.065 | 0.067 | 0.942 | 1.204 | 0.32 |
| Active endocarditis | 1.932 | 0.219 | 1.546 | 2.413 | <0.001 |
| Critical preoperative state | 1.933 | 0.192 | 1.591 | 2.349 | <0.001 |
| Moderate EF (LVEF 31%-50%) | 1.577 | 0.094 | 1.403 | 1.773 | <0.001 |
| Poor EF (LVEF 20%-30%) | 2.314 | 0.226 | 1.911 | 2.802 | <0.001 |
| Very poor EF (LVEF ≤ 20%) | 3.149 | 0.423 | 2.421 | 4.996 | <0.001 |
| MI within previous 90 days | 1.273 | 0.982 | 1.122 | 1.444 | <0.001 |
| Moderate PH (31–55 mmHg) | 1.301 | 0.092 | 1.133 | 1.495 | <0.001 |
| Severe PH (>55 mmHg) | 1.568 | 0.206 | 1.212 | 2.929 | <0.001 |
| Urgent operation | 1.878 | 0.12 | 1.658 | 2.126 | <0.001 |
| Emergency operation | 4.591 | 0.454 | 3.782 | 5.572 | <0.001 |
| Salvage operation | 7.53 | 1.846 | 5.736 | 9.885 | <0.001 |
| Single non-CABG surgery | 1.692 | 0.134 | 1.449 | 1.975 | <0.001 |
| 2 procedures surgery | 2.714 | 0.237 | 2.287 | 3.221 | <0.001 |
| 3 procedures surgery | 4.535 | 0.536 | 3.596 | 5.718 | <0.001 |
| Surgery on thoracic aorta | 1.655 | 0.133 | 1.414 | 1.937 | <0.001 |
| Constant | 0.002 | 0.00003 | 0.001 | 0.082 | <0.001 |

AF, atrial fibrillation; CABG, coronary artery bypass surgery; CI, confidence interval; CCS, Canadian Cardiovascular Society grading of angina pectoris; CPD, chronic pulmonary disease; eGFR, estimated glomerular filtration rate; EF, ejection fraction; IDDM, insulin-dependent diabetes mellitus; LVEF, left ventricular ejection fraction; MI, myocardial infraction; NYHA, New York Heart Association Functional Classification, PH; pulmonary hypertension, OR; odds ratio.

Table S5. Risk factors by multivariate regression model for subgroups. The results show as the odds ratio with 95% confidence interval.

| Risk factors | Low risk  (N=22,479) | Mild risk (N=13,761) | Moderate risk (N=4,715) | High risk (N=1,903) | Very high risk (N=1,314) |
| --- | --- | --- | --- | --- | --- |
| Atrial fibrillation | 0.93 (0.56-1.55), P=0.79 | 0.92 (0.7-1.21), P=0.56 | 0.98 (0.75-1.28), P=0.88 | 1.28 (0.93-1.77), P=0.14 | 1.04 (0.78-1.4), P=0.78 |
| Age (years) | 1.02 (1.01-1.04), P=0.002 | 1.03 (1.01-1.04), P<0.001 | 1.01 (0.99-1.02), P=0.12 | 1 (0.98-1.02), P=0.87 | 1.02 (1.01-1.03), P<0.001 |
| NYHA class II | 0.77 (0.58-1.03), P=0.08 | 0.7 (0.55-0.9), P=0.01 | 0.65 (0.47-0.92), P=0.01 | 0.64 (0.39-1.06), P=0.08 | 0.79 (0.41-1.49), P=0.46 |
| NYHA class III | 1.24 (0.84-1.83), P=0.28 | 0.96 (0.72-1.28), P=0.78 | 0.8 (0.56-1.14), P=0.22 | 0.56 (0.34-0.95), P=0.03 | 0.66 (0.36-1.23), P=0.19 |
| NYHA class IV | 6.37 (2.48-16.4), P<0.001 | 1.82 (1.08-3.06), P=0.02 | 1.21 (0.74-1.99), P=0.45 | 0.7 (0.4-1.25), P=0.23 | 1.29 (0.73-2.27), P=0.39 |
| CCS class IV | 0.47 (0.12-1.89), P=0.29 | 1.47 (0.92-2.36), P=0.11 | 0.88 (0.5-1.55), P=0.67 | 0.98 (0.55-1.72), P=0.93 | 0.78 (0.54-1.13), P=0.19 |
| IDDM | 1.21 (0.69-2.1), P=0.51 | 1.32 (0.99-1.75), P=0.06 | 0.89 (0.63-1.27), P=0.54 | 1.47 (0.99-2.17), P=0.051 | 1.26 (0.91-1.74), P=0.16 |
| Female gender | 1.36 (1.01-1.83), P=0.045 | 1.21 (0.98-1.5), P=0.08 | 1.43 (1.1-1.86), P=0.01 | 1.03 (0.74-1.42), P=0.87 | 1.3 (1.01-1.7), P=0.051 |
| Extracardiac arteriopathy | 1.66 (1.07-2.58), P=0.03 | 1.64 (1.26-2.14), P<0.001 | 1.19 (0.85-1.67), P=0.3 | 0.79 (0.53-1.19), P=0.27 | 1.21 (0.91-1.59), P=0.19 |
| CPD | 1.84 (1.23-2.76), P=0.003 | 1.37 (1.04-1.82), P=0.03 | 1.59 (1.18-2.15), P<0.001 | 1.21 (0.83-1.77), P=0.33 | 1.43 (1.03-2), P=0.04 |
| Poor mobility | 0.95 (0.46-1.96), P=0.89 | 1.4 (1.01-1.94), P=0.049 | 1.23 (0.89-1.7), P=0.21 | 1.23 (0.87-1.75), P=0.24 | 1.36 (1.03-1.78), P=0.03 |
| Previous cardiac surgery | 4.75 (1.44-15.69), P=0.01 | 1.28 (0.72-2.29), P=0.4 | 1.03 (0.56-1.89), P=0.93 | 0.83 (0.42-1.62), P=0.58 | 1.43 (1.04-1.96), P=0.03 |
| Dialysis | 3.17 (0.42-24.04), P=0.26 | 5 (2.88-8.7), P<0.001 | 3.28 (1.55-6.95), P<0.001 | 3.47 (1.56-7.72), P=<0.001 | 1.58 (0.83-3.02), P=0.17 |
| eGFR < 50 ml/min/1.73 m2 | 2.09 (0.62-7.04), P=0.23 | 1.94 (1.31-2.85), P<0.001 | 1.88 (1.13-3.14), P=0.02 | 1.1 (0.6-1.99), P=0.76 | 1.3 (0.91-1.86), P=0.14 |
| eGFR 50-85 ml/min/1.73 m2 | 0.85 (0.62-1.18), P=0.34 | 1.01 (0.8-1.28), P=0.92 | 1.29 (0.93-1.79), P=0.13 | 1.15 (0.76-1.75), P=0.51 | 0.82 (0.58-1.16), P=0.27 |
| Active endocarditis | 2.35 (0.73-7.63), P=0.15 | 2.4 (1.35-4.28), P<0.001 | 1.62 (0.93-2.82), P=0.09 | 1.09 (0.63-1.91), P=0.75 | 1.91 (1.33-2.74), P<0.001 |
| Critical preoperative state | Omitted | 2.63 (0.89-7.81), P=0.08 | 2.04 (1.06-3.91), P=0.03 | 1.33 (0.69-2.58), P=0.39 | 1.58 (1.11-2.25), P=0.01 |
| Moderate EF (LVEF 31%-50%) | 1.55 (1.17-2.06), P=0.003 | 1.87 (1.49-2.34), P<0.001 | 1.48 (1.11-1.97), P=0.01 | 1.08 (0.73-1.59), P=0.72 | 0.86 (0.61-1.21), P=0.39 |
| Poor EF (LVEF 20%-30%) | 5.55 (2.47-12.48), P<0.001 | 1.51 (0.86-2.64), P=0.15 | 1.96 (1.17-3.27), P=0.01 | 1.71 (0.92-3.18), P=0.09 | 1.26 (0.84-1.9), P=0.26 |
| Very poor EF (LVEF ≤ 20%) | 21.68 (8.46-55.53), P<0.001 | 4 (2.08-7.7), P<0.001 | 1.76 (0.86-3.59), P=0.12 | 1.58 (0.73-3.39), P=0.24 | 1.79 (1.1-2.89), P=0.02 |
| MI within previous 90 days | 1.17 (0.83-1.66), P=0.36 | 1.27 (0.99-1.63), P=0.06 | 0.97 (0.72-1.29), P=0.82 | 1.09 (0.77-1.55), P=0.62 | 1.5 (1.1-2.05), P=0.01 |
| Moderate PH (31–55 mmHg) | 1.32 (0.87-1.99), P=0.19 | 1.57 (1.22-2.03), P<0.001 | 1.43 (1.07-1.9), P=0.02 | 1.27 (0.9-1.8), P=0.17 | 0.78 (0.58-1.05), P=0.1 |
| Severe PH (>55 mmHg) | 1.96 (0.45-8.44), P=0.37 | 1.72 (0.85-3.5), P=0.13 | 2.13 (1.28-3.57), P<0.001 | 0.86 (0.47-1.59), P=0.63 | 1.44 (0.98-2.13), P=0.06 |
| Urgent operation | 1.79 (1.29-2.49), P<0.001 | 1.43 (1.12-1.84), P=0.01 | 1.87 (1.38-2.53), P<0.001 | 1.89 (1.28-2.79), P<0.001 | 1.7 (1.09-2.65), P=0.02 |
| Emergency operation | 8.49 (3.8-18.97), P<0.001 | 5.14 (3.41-7.73), P<0.001 | 3.86 (2.39-6.24), P<0.001 | 3.26 (1.83-5.82), P<0.001 | 2.56 (1.57-4.15), P<0.001 |
| Salvage operation | Omitted | 12.68 (3.87-41.48), P<0.001 | 6.11 (2.32-16.06), P<0.001 | 9.02 (3.49-23.35), P<0.001 | 4.26 (2.53-7.15), P<0.001 |
| Single non-CABG surgery | 2.5 (1.84-3.4), P<0.001 | 1.7 (1.29-2.42), P<0.001 | 1.04 (0.72-1.51), P=0.82 | 1.08 (0.66-1.77), P=0.75 | 0.95 (0.61-1.49), P=0.83 |
| 2 procedures surgery | 2.59 (1.84-4.72), P=0.002 | 2.57 (1.81-3.63), P<0.001 | 1.6 (1.02-2.5), P=0.04 | 1.58 (0.88-2.83), P=0.13 | 1.81 (1.13-2.91), P=0.01 |
| 3 procedures surgery | Omitted | 2.9 (1.67-5.35), P<0.001 | 2.37 (1.28-4.42), P=0.006 | 2.19 (0.99-4.83), P=0.052 | 3.21 (1.75-5.87), P<0.001 |
| Surgery on thoracic aorta | 1.61 (0.83-3.13), P=0.16 | 1.42 (0.94-2.14), P=0.09 | 1.55 (1.02-2.36), P=0.04 | 1.01 (0.61-1.67), P=0.97 | 2.01 (1.47-2.76), P<0.001 |
| Constant | 0.004 | 0.01 | 0.01 | 0.06 | 0.11 |

AF, atrial fibrillation; CABG, coronary artery bypass surgery; CCS, Canadian Cardiovascular Society grading of angina pectoris; CPD, chronic pulmonary disease; eGFR, estimated glomerular filtration rate; EF, ejection fraction; IDDM, insulin-dependent diabetes mellitus; LVEF, left ventricular ejection fraction; MI, myocardial infraction; NYHA, New York Heart Association Functional Classification, PH; pulmonary hypertension.

Table S6. Mortality rates in short-, mid- and long-term follow-up.

| EuroSCORE II risk | | In hospital mortality; % (N) | 30-days mortality; % (N) | 90-days mortality; % (N) |
| --- | --- | --- | --- | --- |
| All patients | Low (N=22,479) | 1.1 (249) | 1.6 (349) | 2.2 (492) |
|  | Mild (N=13,761) | 3.4 (469) | 4.6 (631) | 6.3 (866) |
|  | Moderate (N=4,715) | 8 (379) | 9.9 (466) | 12.6 (596) |
|  | High (N=1,903) | 14.2 (270) | 17.4 (332) | 21.9 (417) |
|  | Very high (N=1,314) | 35.7 (469) | 41.2 (541) | 46 (604) |
|  | Total (44,172) | 4.1 (1,830) | 5.2 (2,319) | 6.7 (2,975) |
| AF (+) group | Low (N=1,615) | 1.2 (19) | 2 (33) | 2.9 (47) |
|  | Mild (N=2,260) | 3.3 (75) | 4.8 (108) | 6.7 (152) |
|  | Moderate (N=1,159) | 7.9 (91) | 9.4 (109) | 12.3 (143) |
|  | High (N=523) | 13.6 (71) | 15.9 (83) | 21 (110) |
|  | Very high (N=349) | 33.81 (118) | 39 (136) | 42.7 (149) |
|  | Total (N=5,906) | 6.3 (375) | 7.9 (469) | 10.2 (601) |
| AF (-) group | Low (N=20,864) | 1.1 (230) | 1.5 (316) | 2.1 (445) |
|  | Mild (N=11,501) | 3.4 (393) | 4.5 (523) | 6.2 (714) |
|  | Moderate (N=3,556) | 8.1 (290) | 10 (357) | 12.7 (453) |
|  | High (N=1,380) | 14.4 (199) | 18 (249) | 22.2 (307) |
|  | Very high (N=965) | 36.4 (351) | 42 (405) | 47.2 (455) |
|  | Total (N=38,266) | 3.8 (1,470) | 4.8 (1,850) | 6.2 (2,374) |

Table S7. Summary of calibration performance for total cohort and subgroups

|  | Probability interval | N | Discharged alive | | Died in hospital | | χ2 | P |
| --- | --- | --- | --- | --- | --- | --- | --- | --- |
|  |  |  | O | E | O | E |  |  |
| All patients | 0.499-0.804 | 4,417 | 4,394 | 4,387.8 | 23 | 29.2 | 444.6 | <0.001 |
|  | 0.804-1.045 | 4,418 | 4,383 | 4,375.9 | 34 | 41.1 |  |  |
|  | 1.046-1.296 | 4,417 | 4,375 | 4,365.3 | 42 | 51.7 |  |  |
|  | 1.297-1.594 | 4,417 | 4,355 | 4,353.7 | 62 | 63.3 |  |  |
|  | 1.595-1.961 | 4,417 | 4,340 | 4,339.3 | 77 | 77.7 |  |  |
|  | 1.962-2.469 | 4,417 | 4,320 | 4,320.3 | 97 | 96.7 |  |  |
|  | 2.469-3.216 | 4,417 | 4,275 | 4,292.4 | 142 | 124.6 |  |  |
|  | 3.216-4.552 | 4,417 | 4,232 | 4,248.7 | 185 | 168.3 |  |  |
|  | 4.553-7.843 | 4,417 | 4,103 | 4,155.5 | 314 | 261.5 |  |  |
|  | 7.844-95.080 | 4,419 | 3,565 | 3,562.6 | 854 | 856.4 |  |  |
|  | Total | 44,172 | 42,342 | 40,400,7 | 1830 | 1771,3 |  |  |
| AF (+) group | 0.499-1.167 | 592 | 588 | 586.8 | 4 | 5.2 | 62.7 | <0.001 |
|  | 1.167-1.624 | 592 | 586 | 583.8 | 6 | 8.2 |  |  |
|  | 1.624-2.144 | 592 | 576 | 580.9 | 16 | 11.1 |  |  |
|  | 2.145-2.69 | 592 | 580 | 577.7 | 12 | 14.3 |  |  |
|  | 2.691-3.383 | 592 | 576 | 574.1 | 16 | 17.9 |  |  |
|  | 3.384-4.306 | 592 | 564 | 569.3 | 28 | 22.7 |  |  |
|  | 4.307-5.642 | 592 | 566 | 562.8 | 26 | 29.2 |  |  |
|  | 5.650-7.895 | 592 | 547 | 552.5 | 45 | 39.5 |  |  |
|  | 7.901-13.539 | 592 | 529 | 531.5 | 63 | 60.5 |  |  |
|  | 13.57-93.779 | 578 | 420 | 409.9 | 158 | 168.1 |  |  |
|  | Total | 5,906 | 5,532 | 5,529.3 | 374 | 376,7 |  |  |
| AF (-) group | 0.499-1.167 | 3,826 | 3,807 | 3,801.2 | 19 | 24.8 | 363.2 | <0.001 |
|  | 0.78-1 | 3,826 | 3,801 | 3,791.9 | 25 | 34.1 |  |  |
|  | 1-1.237 | 3,826 | 3,789 | 3,783.2 | 37 | 42.8 |  |  |
|  | 1.237-1.502 | 3,826 | 3,778 | 3,773.8 | 48 | 52.2 |  |  |
|  | 1.503-1.819 | 3,826 | 3,763 | 3,762.8 | 63 | 63.2 |  |  |
|  | 1.820-2.244 | 3,826 | 3,747 | 3,748.7 | 79 | 77.3 |  |  |
|  | 2.245-2.909 | 3,826 | 3,717 | 3,728 | 109 | 98 |  |  |
|  | 2.91-4.047 | 3,826 | 3,678 | 3,695.2 | 148 | 130.8 |  |  |
|  | 4.048-6.95 | 3,826 | 3,581 | 3,625.8 | 245 | 200.2 |  |  |
|  | 6.95-95.080 | 3,832 | 3,149 | 3,161.2 | 683 | 670.8 |  |  |
|  | Total | 38,266 | 36,810 | 36,871.8 | 1,456 | 1394.2 |  |  |

AF, atrial fibrillation; E, expected; N, number; O, observed;
